# From Mind to Mouth: Understanding Active Publics in China and Their Communicative Behaviors on GM Foods

**DOI:** 10.3390/ijerph20010578

**Published:** 2022-12-29

**Authors:** Myoung-Gi Chon, Linjia Xu, Jiaying Liu, Jeong-Nam Kim, Jarim Kim

**Affiliations:** 1 School of Communication and Journalism, Auburn University, Auburn, AL 36849-5206, USA; 2 School of Chinese Language and Literature, University of International Business and Economics, Beijing 100029, China; 3 Department of Communication Studies, University of Georgia, Athens, GA 30602-2607, USA; 4 Gaylord College of Journalism & Mass Communication, University of Oklahoma, Norman, OK 73019-4201, USA; 5 Department of Communication, Yonsei University, Seoul 03722, Republic of Korea

**Keywords:** genetically modified foods, communicative behaviors, public segmentation, situational theory of problem solving

## Abstract

Using an online survey conducted in China (N = 1089), this study aims to understand the characteristics of active publics on the issue of genetically modified (GM) foods and provide effective communication strategies with active publics in China. In doing so, this study segments active publics regarding GM foods and predicts their communicative behaviors on GM foods by using the theoretical framework of situational theory of problem solving (STOPS). The results of the study revealed the demographic characteristics of active publics, situational, and media factors to predict information seeking, forefending, and forwarding about GM foods. Theoretical and practical implications of the findings are discussed.

## 1. Introduction

Genetically modified (GM) foods have been a controversial topic worldwide over the past decade. According to recent research [1], two-fifths (39%) of Americans perceive GM foods to be worse for their health than other foods. Negative claims about GM foods’ effects on human health have raised even more concerns outside of the U.S. For example, in Asia, 41.4% of Chinese oppose GM foods whereas only 11.9% support GM foods and 46.7% are neutral [2]. Public acceptance of GM foods has led scholars’ attention [3].

Given the increasing negative attitudes about GM foods in China, it is essential for public health organizations to research why individuals are concerned about the issue by identifying active publics on the issue of GM foods. Public relations scholars suggest public segmentation as a first step for organizations to develop strategies to solve an organizational problem [4,5]. While segmenting active publics rather than targeting the general population regarding health issues, organizations can increase their effectiveness in reaching and communicating with target publics [6]. According to Grunig and Kim [6], active publics refers to a group of people who recognize a given issue and take actions to change the issue through communication behaviors.

Despite the importance of understanding active publics and communicative behaviors, there are very few studies of public segmentation in relation to GM foods that offer public health organizations ways to communicate with key publics and plan strategies to deal with potential issues in China. The purpose of this study is twofold. First, by using a theoretical framework of situational theory of problem solving (STOPS), it aims to segment active publics regarding GM foods in China [5]. Secondly, this study aims to predict communicative behaviors of active publics toward GM foods. The results of this study will contribute to public relations campaigns and risk–health communication regarding GM food issues.

## 2. Literature Review

### 2.1. The Issue of GM Foods in China

China was among the first countries to commercialize GM crops and has invested substantial efforts in the development and application of GM technologies in the food industry [7]. GM foods and consumers’ right to know about GM products and ingredients have been a public issue since 2000 in China [8]. In recent years, food-related issues have received increasing media coverage and scholarly attention [9]. Online debates over GM foods between two Chinese celebrities have especially caught the public’s attention. A famous TV host, Yongyuan Cui, opposed GM foods, while a famous science writer, Zhouzi Fang, supported them. Cui produced and widely circulated a documentary film about the dangers of GM foods, demonstrating how GM technology was controversial outside of China. As a result, the percentage of Chinese citizens who consider GM foods safe has dramatically decreased from 35% in 2002 to 13% in 2012, according to a national survey [10]. Most Chinese people today hold somewhat skeptical attitudes towards GM foods. According to a national survey conducted in 2016, 41.4% of Chinese oppose GM foods and 46.7% have a neutral attitude [2].

Despite the general skepticism toward GM foods, the consumption of such foods, particularly GM soybeans has been steadily increasing in China these years [11]. Such an interesting, contradictory pattern is likely attributed to the lack of awareness, knowledge and understanding of GM-foods-related issues among Chinese consumers, especially some subgroups of this population. In fact, recent studies observed quite an amount of heterogeneity among the Chinese population regarding their knowledge, belief, attitude, and downstream behaviors related to GM food purchase, advocacy/opposition stance, and public policy support decisions [12,13]. For example, through a large-scale survey conducted across 11 cities in China, Zhang and colleagues (2010) concluded that Chinese consumers’ perceptions and attitudes toward GM-foods-related risks and benefits emanate from various individual difference factors including their social demographics, trust in government/institutions, lifestyles, etc. They thus suggested that modern Chinese consumers consist of sophisticated clusters, exhibiting distinct attitudes, behavior patterns and tendencies to act on what they believe or support.

Although scholars in China have examined public concerns around GM foods, few have paid attention to strategic and effective communication through public segmentation. In fact, treating all populations as an equal mass inhibits effective understanding of civic and social dynamics [6]. As the issue has raised concerns in China, the largest population in the world, it is critical to investigate the issue of GM food by understanding active publics on the issue.

### 2.2. Public Segmentation to Identify Active Publics on the Issue of GM Foods

The basic idea of segmentation is to “divide a population, market, or audience into groups whose members are more like each other than members of other segments” [14]. In public relations, public segmentation is useful in identifying key publics in a problematic situation and building relationships with publics [6]. As each segment of publics have different needs, it is imperative for an organization to target each segment through different communication strategies [4]. While segmentation in marketing is intended to enhance cost effectiveness in promoting products or services, segmentation in public relations is intended to decrease the cost of problem solving and increase publics’ support and cost effectiveness [15].

The term “public” is popular in public opinion, political science, public affairs, and public relations [6]. Given the concept of “public,” scholars in public relations generally define “public” as “a homogeneous social collectivity who identify a similar problem and work together toward problem resolution” [16]. In addition, the concept of “publics” influences the understanding of public opinion [17]. Survey research tends to measure mass opinion, viewing public opinion as the aggregate opinion of everyone. That is, the concept of “publics” is helpful to distinguish public opinion from the crowd, the public, and the mass [6]. When it comes to GM foods, there are multiple issues and people differently perceive the significance of those issues.

### 2.3. Summation Method Using the Theoretical Framework of STOPS

STOPS provides a conceptual frame and summation method to classify the general population into strategic subgroups [5]. STOPS was proposed to expand and generalize the situational theory of publics (STP) [6]. While STP conceptually posited a person as an economic man facing a decision situation, STOPS shifted the focus of STP from a decision-making process to a problem-solving process. STOPS explains the communicative action of publics in terms of three domains: information acquisition (i.e., information seeking and information attending), information selection (i.e., information forefending and information permitting), and information transmission (i.e., information forwarding and information sharing) as dependent variables.

Three situational and perceptual variables (i.e., problem recognition, involvement recognition, and constraint recognition) are used in segmenting publics [5,14]. Problem recognition is defined as “one’s perception that something is missing and that there is no immediately applicable solution to it” [6]. According to Grunig [18], a person enters a problematic situation when he or she perceives a problem and is not able to find an immediate solution. Another perceptual variable, involvement recognition, refers to the extent to which the connection with a problem influences how much they think about the problem. Lastly, constraint recognition refers to the extent of perceiving obstacles in a situation [6]. Unlike problem recognition and involvement recognition, constraint recognition discourages the communicative action of publics in a problematic situation. In the current study, we used the reversed items for constraint recognition.

The summation method using situational variables helps classify the general population into four subtypes of publics who face the same problem: an active public, an aware public, a latent public, and a nonpublic [14]. In public relations research, summation method means public segmentation method by using three situational variables such as problem recognition, constraint recognition, and involvement recognition to identify four types of publics (i.e., active, aware, latent, and non-public) [5]. This is based on an assumption that publics are active in communication behaviors when they are highly engaged in problem solving [6]. A nonpublic is a group who are not identified by the three conditions. A latent public is a group who face a similar problem but do not detect a problem. When they recognize a problem, they become an aware public. Finally, an active public faces a problem, perceives the significance of the issue, and organizes to do something about it. Situational theories predict that an active public is more likely to take, select, and transmit information in a problematic situation than other types of publics. In the context of risk-crisis communication, an active public is a key public who needs strategic communication from an organization [19].

This study identifies four types of publics by using situational theory (i.e., summation method). After segmenting four types of public to identify active publics, this study attempts to understand the characteristics of active publics by comparing demographic variables such as gender, age, education, and political stance in China. When considering that treating general population leads to trouble understanding the issue of GM foods and social dynamics, it is essential to segment publics in a certain situation. We propose the following research questions:

**RQ1:**  How are publics segmented in China on the issue of GM foods?

**RQ2:**  What are the characteristics of active publics according to their gender, age, education, and political stance in China?

In this study, we propose communicative behaviors of active publics as outcomes of problem-solving behaviors toward the issue. Using a framework of STOPS, we attempt to predict communicative behaviors of active publics in China. In STOPS, communicative action in problem solving explains individuals’ activeness in communication behaviors such as information acquisition, information selection, and information transmission. Each of these three dimensions have active and passive variables. Three domains of communicative action have six variables: information seeking (active) and information attending (passive) in the information acquisition domain, information forefending (active) and information permitting (passive) in the information selection domain, and information forwarding (active) and information sharing (passive) in the information transmission domain.

According to STOPS, individuals are active in communicative action in problem solving when they have high problem recognition and involvement recognition and less constraint recognition regarding a given issue [20]. As active communication behaviors, Kim and Grunig conceptualized information seeking as “the planned scanning of environment for messages about a specified topic” and information forefending as “the extent to which a communicator fends off certain information in advance by judging its value and relevance for a given problem-solving task.” They also conceptualized information forwarding as “a planned, self-propelled information giving to others” [6]. Given the assumption of STOPS theory, we propose the following hypotheses:

**H1:**  Problem recognition, constraint recognition, and involvement recognition of active publics in China are positively associated with information seeking on the issue of GM foods.

**H2:**  Problem recognition, constraint recognition, and involvement recognition of active publics in China are positively associated with information forefending on the issue of GM foods.

**H3:**  Problem recognition, constraint recognition, and involvement recognition of active publics in China are positively associated with information forwarding on the issue of GM foods.

With the prediction of three active communication behaviors of active publics on the issue of GM foods, the association between media channels and communicative behaviors will provide a more sophisticated understanding of active publics and help health organizations develop effective health campaigns. Using media usage variables, prior research attempted to segment publics to predict individuals’ health behaviors [21]. Because there is little existing research on how media use may influence the communicative actions of publics, we propose the following research questions instead of hypotheses:

**RQ3:**  How is using the Internet, television, and newspaper associated with active communication behaviors on the issue of GM food in China?

## 3. Materials and Method

### 3.1. Data Collection and Participants

Using nonprobability quota sampling, we conducted an online survey in China to examine the perceptions of GM food issues of Chinese publics. Although quota sampling is not probability sampling, it allowed us to sample respondents proportionate to the distributions of traits and characteristics of Chinese netizens who formed the most important subgroup engaging in communicative actions about scientific issues in China. The final sample size used for analysis was 1089. Table 1 shows the participants were from a wide range of age groups, whose distribution is comparable to that of the Chinese netizen sample reported in the most recent censuses conducted in China [22], suggesting that our sample is more indicative of opinions and ideas of Chinese netizens, rather than the general population. The profile of the SoJump panelists is representative of Chinese netizens and reflects their patterns in social media use [23].

The SoJump research firm, which is one of China’s largest survey companies [24], sent survey invitations to its panel participants who met the criteria. If the panelists decided to participate in a study, they received immediate compensation. To ensure data quality, the study used Sojump’s paid sample service, which possesses a panel of more than 2.6 million respondents from different cities in China. The respondents receive immediate reimbursement (points redeemable for cash) once they complete the study. To ensure answer quality for this study, SoJump added in foil questions and manually checked a random sample of answers from the participants. This study has passed the research ethics review in the authors’ institution.

The data were collected from January to February 2018 through the SoJump online survey firm, with its nationwide sample pool of 2.6 million respondents. As shown in Table 1, the total sample size for data analysis was 1089, with 49.9% (*n* = 543) male participants and 50.1% (*n* = 546) female participants. Of the participants, 36.3% (*n* = 395) indicated that they were 18–29 years old, 43.2% (*n* = 470) were 30–39 years old, 14.2% (*n* = 155) were 40–49 years old, 5.1% (*n* = 56) were 50–59 years, and 1.2% (*n* = 13) were more than 60 years old.

### 3.2. Measures

Situational variables and communicative behaviors variables were adopted from previous studies [20]. Respondents were asked questions about what they thought about GM foods, ranked on a five-point scale (ranging from 1 = strongly disagree to 5 = strongly agree).

Two items in the survey were used to measure situational variables of problem recognition (*M* = 3.86, *SD* = 0.79, *r* = 0.51), involvement recognition (*M* = 4.08, *SD* = 0.71, *r* = 0.55), and constraint recognition (*M* = 3.53, *SD* = 0.82, *r* = 0.50). Pearson correlations of three variables are as follows. Problem recognition and constraint recognition (0.374, *p* < 0.01). Problem recognition and involvement recognition (0.423, *p* < 0.01). Constraint recognition and involvement recognition (0.325, *p* < 0.01). In this study, we used the reversed items for constraint recognition. Example items of situational variables are as follows. Problem recognition (e.g., I think GM food is a serious social problem), constraint recognition (e.g., I feel that my idea or opinion matters to those who are addressing the GM food issue in government or corporations), and involvement recognition (e.g., I see a close connection between my life and the issue).

To measure communicative behaviors of publics on the GM food issue, we also adopted variables from previous research [20]. According to STOPS, active communication behaviors in three dimensions of communicative action are as follows: information seeking (e.g., I search for information about the GM food issue on the Internet), information forefending (I can easily tell reliable information about the GM food issue), and information forwarding (e.g., if possible, I take time to explain this problem to friends or family members). Three variables were used to measure communicative behaviors regarding the GM food issue: information seeking (*M* = 3.81, *SD* = 0.81, *α* = 0.80), information forefending (*M* = 3.15, *SD* = 0.68, *α* = 0.54), and information forwarding (*M* = 3.69, *SD* = 0.71, *α* = 0.70).

Regarding media usage, using a five-point scale (1 = never, 2 = less than once a week, 3 = 3–5 times a week, 4 = once a day, 5 = more than 3–5 times a day), respondents rated how often they use the Internet (*M* = 3.87, *SD* = 0.94), television (*M* = 3.33, *SD* = 1.02) and newspaper (*M* = 2.71, *SD* = 1.13) to obtain information about GM food or science knowledge. The unit of analysis of this variable is to measure frequencies to use media.

Using the summation method [5], we segmented participants into four types of publics. Three situational variables (i.e., problem recognition, constraint recognition, and involvement recognition) were used to identify four types of publics (i.e., active public, aware public, latent public, and nonpublic) regarding the GM food issue. In particular, the survey items measuring three situational variables were re-coded into two scales, 0 (=low) and 1 (=high) after taking the midpoint of the survey scale. The points ranged from 0 to 3 when the three variables were added (e.g., 3 = active public, 2 = aware public, 1 = latent public, and 0 = nonpublic).

## 4. Results

Using the summation method, we identified four types of publics among Chinese netizens (N = 1089). Table 2 shows the general results of public segmentation on the GM food issue. Of the 1089 total participants, 50.8% (*n* = 553) were classified as active public, 32.3% (*n* = 352) as aware public, 13.1% (*n* = 143) as latent public, and 3.8% (*n* = 41) as nonpublic.

To understand the characteristics of active publics on the GM issue in China, first, we obtained descriptive statistics by using cross tabulation analysis. Table 3, Table 4, Table 5 and Table 6 present the distribution of gender, age, education, and political stance (i.e., descriptive statistics) among active publics. First, Table 3 shows the relationship between publics and gender. Second, Table 4 indicates different age group in four types of publics. The 30–39 age group had the highest proportion of active publics (47.9%, *n* = 265) regarding the GM food issue in China. Third, looking at the relationship between active publics and level of education (see Table 5), those who completed university were the most active public (81.0%, *n* = 866) in China. Fourth, in terms of political stance (see Table 6), China has the highest ratio of active public among independent (69.6%, *n* = 385) and neutral groups (45.5%, *n* = 96) in terms of political stance. The result shows that partisanship is not a critical factor that has an impact on individuals’ activeness to perceive the GM food issue.

After identifying active publics on the GM food issue, this study examined how situational variables and media variables predict three communicative behaviors. To test the hypotheses and explore the research questions, ordinary least squares (OLS) multiple regressions were performed in which demographic variables (i.e., gender and age), situational variables (i.e., problem recognition, constraint recognition, involvement recognition), and media variables (i.e., Internet, television, and newspaper) were entered. The dependent variables were information seeking, information forefending, and information forwarding (See Table 7).

Using SPSS program 27, first of all, we conducted regression analyses of data without outliers. Moreover, this study checked multicollinearity for the OLS regressions. Assumptions were checked to ensure that there was no violation. In checking multicollinearity, the variance inflation factor (VIF) and tolerance showed that there was no violation of multicollinearity in all independent variables. (e.g., VIF of each variable < 10 and tolerance of each variable > 0.10). Regarding effect size, we reported 0.61 (information seeking), 0.33 (information forefending), and 0.52 (information forwarding) by using Cohen’s F which indicates 10 = small effect size, 25 = medium effect size, and 40 = large effect size.

H1 predicted that situational variables would be positively associated with information seeking in China. As shown in Table 7, involvement recognition was the strongest predictor of information seeking (*β* = 0.32, *p* < 0.001). The results also show that problem recognition was positively related to information seeking (*β* = 0.12, *p* < 0.01). However, constraint recognition was not significantly associated with information seeking. After controlling for demographic variables and situational variables, Internet use was significantly and positively associated with information seeking (*β* = 0.17, *p* < 0.001). Newspaper use was also positively associated with information seeking (*β* = 0.18, *p* < 0.001).

H2 predicted that situational variables would be positively related to information forefending in China. As shown in Table 7, constraint recognition was positively associated with information forefending (*β* = 0.20, *p* < 0.001). Problem recognition was also positively related to information forefending (*β* = 0.10, *p* < 0.05). However, involvement recognition was not associated with information forefending. Controlling demographic variables and situational variables, Internet use was significantly and negatively associated with information forefending (*β* = −0.10, *p* < 0.05). Newspaper use was also positively related to information seeking (*β* = 0.15, *p* < 0.01). Similar to information seeking, Internet and newspaper use were positively associated with information forefending.

H3 predicted that situational variables would be positively associated with information forwarding in China. As Table 7 shows, all situational variables were positively associated with information forwarding. Specifically, problem recognition (*β* = 0.14, *p* < 0.01), constraint recognition (*β* = 0.16, *p* < 0.001), and involvement recognition (*β* = 0.14, *p* < 0.01) were statistically associated with information forwarding. Regarding the relationship between media variables and communicative behaviors, the results found that Internet (*β* = 0.17, *p* < 0.001) and newspaper use (*β* = 0.18, *p* < 0.001) were positively related to information seeking. Internet (*β* = −0.10, *p* < 0.05) and newspaper use (*β* = 0.15, *p* < 0.01) were associated with information forefending. Finally, Internet (*β* = 0.15, *p* < 0.001) and newspaper use (*β* = 0.16, *p* < 0.001) were positively related to forwarding.

## 5. Discussion

Our study on the public perception of the issue of GM foods in China maps out the social topography and significant resources to predict active publics’ communicative behaviors on GM food issues. This study illustrates the steps and grounds for a collaborative, deliberative social process on the controversial and yet to be agreed on issue. Understanding what citizens and GM food publics in China fear and want is the key task for politicians, government, corporations, and scientists. The current study presents the first lead look for these large-scale actors and their profiles through the lens of STOPS on food safety issues by identifying active publics and their communicative behaviors. The findings of our study provide a theory-based identification of strategic constituencies on the issue of GM foods in China.

This study contributes to the prior literature on public segmentation by applying the summation method to GM food issues and identifying the characteristics of active publics related to the GM food issue in China. Since treating all populations as an equal mass makes it difficult to understand civic and social dynamics effectively, the identification of active publics on the GM issues contributes to the success of public relations campaign [6,15]. In particular, the results of this study driven by the summation method provide characteristics of active publics on GM food issues across China. In general, Chinese people are actively involved in the issue of GM and perceive fewer obstacles to solve the issue. In particular, 30–39-year-olds with high education levels are an important target-public for health organizations in China. As the issue of GM foods in China has become a critical social issue, it is necessary for governmental and social institutions to understand how and why people view GM foods that way, how they redefine the problem and causes, and what they demand to be done, foretelling what will eventually be major concerns and perceptions regarding the issue. The findings of this study allow public relations practitioners working on GM foods issues to better understand active publics and effectively communicate with active publics through a more audience-focused campaign plan.

In addition, using data of active publics, we examined how situational variables and media variables predict active communication behaviors such as information seeking, information forefending, and information forwarding. According to STOPS, individuals are active in communication when they are engaged in their problems [20]. Indeed, three communication behaviors became indicators to explain when and why active publics attempt to seek and forward GM foods information as well as fend off the information.

Problem recognition in China consistently predicts the three communication behaviors of the GM food issue. When people perceive the issue of GM food but feel incapable of finding an immediate solution to the issue, they are more active in communication behaviors to seek, forefend, and forward the issue of GM foods. Interestingly, constraint recognition, which was reversely recoded in the present study, was positively associated with both information forefending and forwarding. In other words, Chinese people in current society are less likely to perceive obstacles or barriers to make a difference on GM foods issues.

Active publics on GM food issues tend to fend off GM information as well as forward the information. According to the China Internet Network Information Center, 97% of Chinese netizens are currently using social media such as Wechat and Weibo, with the primary motivation to stay connected with families and friends, and social media has become the most important information source for Chinese people other than traditional media [25]. The most commonly used social media (by 87% of netizens) is WeChat, a “super-app” that allows for instant messaging and information sharing with families, friends, and provides all types of news and information including science and health related topics [25].

Interestingly, Internet use is a consistent factor to predict the three communicative behaviors, especially in China. Chinese people are more likely to seek and forward the issue of GM food via the Internet. Chinese people tend to fend off GM information when they read newspapers; they are less likely to fend off information of GM foods through the Internet. As the Internet provides an interactive communication environment between an organization and its publics, it is more expected to decrease misinformation of the GM food issues and increase campaign effects. Therefore, the Internet is a critical medium to communicate with active publics in China.

Constraint recognition and Internet usage are positively associated with information seeking in China. When people do not feel constrained, they are more likely to seek information about GM food issues through the Internet. Again, the Internet is a critical channel to communicate with active publics in China. In the digital age, individuals are not only receivers of messages about GM food issues but also senders of information about the issue through social media. From the perspective of health organizations in China, social media are strategic channels to communicate with active publics on the issue of GM foods. More importantly, the results of this study found that using the Internet decreases information forefending on GM foods. Information forefending in communicative action of problem solving refers to “a problem solver’s tendency to fend off certain information by judging its value and relevance in advance in a given problem-solving task” [26]. Accordingly, for public relations communicators in the public sector, the Internet could be an effective tool to decrease misinformation and increase communication efficiency about GM food issues.

Interestingly, newspaper use is strongly associated with information forefending of active publics about the issue. This finding suggests that Chinese people who use newspapers to obtain information are more likely to fend off GM food information in advance based on their experiences or knowledge related to the issue. That is, the different results contribute to practical implications to show how media are differently used in communication strategies between government and its publics on a given issue. The problem for individuals is not the lack of information seeking but the strong presence of information forefending [20]. The results of this study clearly show that the Internet can be a strategic tool, rather than newspapers, to communicate with publics via health campaign messages.

STOPS defines concerned citizens as problem solvers arising around the problematic situation [6]. Likewise, experts, politicians, or governments who deal with the issue are active problem solvers who create products, formulate policies and regulations, and often patrol public responses. Both entities are highly motivated to learn, select, and talk about causes of the problem and the issue. Accordingly, it is meaningful to use a conceptual frame to see and capture both problem-solving groups and their cognitive and perceptual status in the evolutionary trajectory of the issue of GM foods.

Finally, this study suggests the importance of a cumulative process for mutual learning and negotiation between two entities such as active publics and organizations (e.g., government and science/technology industry) dealing with GM food issues to reach “agreements” on the GM food issue and achieve solutions (see the more systematic discussion and prescription) [16]. When it comes to a controversial issue, the two entities should take time to “agree” by using two-way symmetrical communication based on mutual understanding to accelerate a problem resolution [18]. Using two-way symmetrical communication could increase the accuracy and understanding of information and gradually facilitate social agreement; therefore, we believe it would be effective to solve the GM food issues in China.

This study has several limitations. This study used a nonprobability sample based on an online panel, which limits statistical generalization. Future research is encouraged to apply probability sampling to collect more representative data. Using the results of this study, future research may consider new strategies to communicate with active publics on the issue of GM foods in the context of health campaigns. As this study focused on public segmentation and communicative behaviors, future research should consider other factors to better understand active publics’ psychological factors underlying behaviors. For instance, it is possible to test subjective norms, attitudes on GM foods, and self-efficacy to change GM food issues. In addition, it is possible to study how to make nonpublics who are not interested regarding a certain issue among four types of publics active in GM foods and then understand GM issues by decreasing misinformation. The role of public communication to narrow down different perceptions between lay-publics (i.e., general population) and experts in science will be critical by decreasing mis/disinformation regarding the GM food issues in China.

## 6. Conclusions

The study used an online survey conducted in China, aiming to understand the characteristics of active publics on the issue of GM foods and provide effective communication strategies with active publics in China. This study segments active publics regarding GM foods and predicts their communicative behaviors on GM foods by using the theoretical framework of STOPS. The results of the study revealed the characteristics of active publics and examined how situational variables and media variables predict three communicative behaviors. Consequently, our study on the public perception of the issue of GM foods in China maps out the social-topography and significant resources to predict active publics’ communicative behaviors on GM food issues. Particularly, this study illustrates the steps and grounds for a collaborative, deliberative social process on the controversial and yet to be agreed on issue. Understanding what citizens and GM food publics in China fear and want is the key task for politicians, government, corporations, and scientists.

## Figures and Tables

**Table 1:** Sample Descriptive Statistics (N = 1089).

Characteristics	Variables	N (%)
Gender	Male	543 (49.9)
	Female	546 (50.1)
Age	18 to 29	395 (36.3)
	30 to 39	470 (43.2)
	40 to 49	155 (14.2)
	50 to 59	56 (5.1)
	Above 60	13 (1.2)
Education	Above Graduate school	86 (7.9)
	Completed University	866 (80)
	Completed High School	123 (11)
	Below High School	14 (1)
Political Stance	Communist Party/Conservative	289 (26.5)
	Other Parties/Neutral Independent/Liberal	24 (2.2) 776 (71.3)

**Table 2:** Four types of publics in China about the issue of GM foods.

Type of Publics	N (%)
Nonpublic	41 (3.8)
Latent public	143 (13.1)
Aware public	352 (32.3)
Active public	553 (50.8)
Total	1089 (100)

**Table 3:** Four types of publics and gender in China.

	Male	Female
	*n* (%)	*n* (%)
Nonpublic	18 (43.9)	23 (56.1)
Latent public	69 (48.3)	74 (51.7)
Aware public	182 (51.7)	170 (48.3)
Active public	274 (49.5)	279 (50.5)
Total	543 (49.9)	546 (50.1)

**Table 4:** Four types of publics and age in China.

	18–29 *n* (%)	30–39 *n* (%)	40–49 *n* (%)	50–59 *n* (%)	60 *n* (%)
Nonpublic	16 (39.0)	17 (41.5)	7 (17.1)	1 (2.4)	0 (0)
Latent public	70 (49.0)	51 (35.7)	13 (9.1)	9 (6.3)	0 (0)
Aware public	140 (39.8)	137 (38.9)	55 (15.6)	17 (4.8)	3 (0.9)
Active public	169 (30.6)	265 (47.9)	80 (14.5)	29 (5.2)	10 (1.8)
Total	395 (36.3)	470 (43.2)	155 (14.2)	56 (5.1)	13 (1.2)

**Table 5:** Four types of publics and education in China *.

	Above Graduate School *n* (%)	Completed Univ. *n* (%)	Completed High School *n* (%)	Below High School *n* (%)
Nonpublic	2 (4.9)	32 (78.0)	5 (12.2)	2 (4.9)
Latent public	12 (8.4)	106 (74.1)	22 (15.4)	3 (2.1)
Aware public	33 (9.4)	280 (79.5)	34 (9.7)	5 (1.4)
Active public	39 (7.1)	448 (81.0)	62 (11.2)	4 (0.7)
Total	86 (7.9)	866 (79.5)	123 (11.3)	14 (1.3)

* Above graduate school = completed some postgraduate degree/program; Completed Univ. = completed some postgraduate degree/program + attending/dropping out postgraduate program; Completed high school = attending/dropping out university/college + completed high school; Below high school = attending/dropping out high school + completed junior high school + completed elementary(primary) school.

**Table 6:** Four types of publics and political stance in China.

	Communist *n* (%)	Others *n* (%)	Independent *n* (%)
Nonpublic	7 (17.1)	0 (0)	34 (82.9)
Latent public	43 (30.1)	5 (3.5)	95 (66.4)
Aware public	86 (22.4)	4 (1.1)	262 (74.4)
Active public	153 (27.7)	15 (2.7)	385 (69.6)
Total	289 (26.5)	24 (2.2)	776 (71.3)

**Table 7:** Using active publics about the issue of GM foods, multiple regression analysis predicting information seeking, information forefending, and information forwarding in China (N = 553).

Variables	Information Seeking	Information Forefending	Information Forwarding
**Demographic variables**			
Gender ^a^	0.09 *	0.10 *	0.07
Age	0.04	−0.01	−0.06
**Situational variables**			
Problem recognition	0.12 **	0.10 *	0.14 **
Constraint recognition ^b^	0.03	0.20 ***	0.16 ***
Involvement recognition	0.32 ***	−0.06	0.14 **
**Media variables ^c^**			
Internet	0.17 ***	−0.10 *	0.15 ***
Television	0.05	0.05	0.04
Newspaper	0.18 ***	0.15 **	0.16 ***
*R* ^2^	0.27	0.10	0.21
*F* value	24.61	7.88	18.02

* *p* < 0.05 ** *p* < 0.01, *** *p* <.001. ^a^ Gender (Male = 1, female = 0). ^b^ This study used the reversed items for constraint recognition. ^c^ Media use is related to science topic or GM foods.

## Data Availability

The data presented in this study are available on request from the corresponding author. The data are not publicly available due to privacy reasons.

## References

[1] Pew Research Center The New Food Fights: U.S.Public Divides over Food Science. https://www.pewresearch.org/science/2016/12/01/the-new-food-fights/.

[2] Cui K., Shoemaker S.P. (2018). Public perception of genetically-modified (GM) food: A Nationwide Chinese Consumer Study. NPJ Sci. Food.

[3] Raza S.H., Zaman U., Ferreira P., Farías P. (2021). An experimental evidence on public acceptance of genetically modified food through advertisement framing on health and environmental benefits, objective knowledge, and risk reduction. Int. J. Environ. Res. Public Health.

[4] Grunig J.E., Repper F.C., Grunig J.E. (1992). Strategic management, publics, and issues. Excellence in Public Relations and Communication Management.

[5] Kim J.-N. (2011). Public segmentation using situational theory of problem solving: Illustrating summation method and testing segmented public profiles. Prism.

[6] Grunig J.E., Kim J.-N. (2017). Publics approaches to health and risk message design and processing. Oxf. Res. Encycl. Commun..

[7] Hu R., Cai J., Huang J., Wang X. (2012). Silos hamstring Chinese plant biotech sector. Nat. Biotechnol..

[8] Jia H., Fan J. (2015). Why genetically modified crops are resisted- A systematic review of science communication studies. Stud. Sci. Pop..

[9] Wu L., Zhong Y., Shan L. (2013). Influencing factors of public’s risk perception towards food additives. Chin. Rural. Econ..

[10] Peng B., Uang J. (2015). Chinese Consumers’ Knowledge and Acceptances of Genetically Modified Food. Agric. Econ. Manag..

[11] Zhao Y., Deng H., Yu C., Hu R. (2019). The Chinese public’s awareness and attitudes toward genetically modified foods with different labeling. NPJ Sci. Food.

[12] Huang J., Qiu H., Bai J., Pray C. (2006). Awareness, acceptance of and willingness to buy genetically modified foods in Urban China. Appetite.

[13] Zhang X., Huang J., Qiu H., Huang Z. (2010). A consumer segmentation study with regards to genetically modified food in urban China. Food Policy.

[14] Grunig J.E., Salmon C.T. (1989). Publics: Audiences and market segments: Segmentation principles for campaigns. Information Campaigns: Balancing Social Values and Social Change.

[15] Kim J.-N., Ni L., Sha B.L. (2008). Breaking down the stakeholder environment: Explicating approaches to the segmentation of publics for public relations research. J. Mass Commun. Q..

[16] Kim J.-N., Ni L. (2013). Integrating formative and evaluative research in two types of public relations problems: A review of research programs within the behavioral, strategic management paradigm. J. Public Relat. Res..

[17] Price V. (1992). Public Opinion.

[18] Grunig J.E., Heath R.L. (2001). Two-way symmetrical public relations: Past, present, and future. Handbook of Public Relations.

[19] Kim Y., Miller A., Chon M.G. (2016). Communicating with key publics in crisis communication: The synthetic approach to the public segmentation in CAPS (Communicative Action in Problem Solving). J. Contingencies Crisis Manag..

[20] Kim J.-N., Grunig J.E. (2011). Problem solving and communicative action: A situational theory of problem solving. J. Commun..

[21] Rodgers S., Chen Q., Duffy M., Fleming K. (2007). Media usage as health segmentation variables. J. Health Commun..

[22] The Population Census Office of the State Council The 42nd China Statistical Report on Internet Development. http://www.cnnic.net.cn/hlwfzyj/hlwxzbg/hlwtjbg/201808/P020180820630889299840.pdf.

[23] Zhou Z., Wu J.P., Zhang Q., Xu S. (2013). Transforming visitors into members in online brand communities: Evidence from China. J. Bus. Res..

[24] Yang Y., Liu X., Fang Y., Hong Y. (2014). Unresolved World War II animosity dampens empathy toward 2011 Japanese earthquake and tsunami. J. Cross-Cult. Psychol..

[25] The China Internet Network Information Center The 44th China Statistical Report on Internet Development. http://www.cnnic.cn/hlwfzyj/hlwxzbg/hlwtjbg/201908/P020190830356787490958.pdf/.

[26] Kim M.H., Jung M.A., Park S.W., Kim Y.S., Kyung K.H. (2001). Survey of consumer awareness and attitudes about food biotechnology in Korea. J. Korean Soc. Food Hyg. Saf..

